# Early versus delayed computed tomography-guided celiac plexus neurolysis for palliative pain management in patients with advanced pancreatic cancer: a retrospective cohort study

**DOI:** 10.3389/fneur.2023.1292758

**Published:** 2023-11-07

**Authors:** Fan Lu, Xiaojia Wang, Jie Tian, Xuehan Li

**Affiliations:** ^1^Department of Pain Management, West China Hospital, Sichuan University, Chengdu, China; ^2^Department of Anesthesiology, Sichuan Cancer Hospital and Institute, Sichuan Cancer Center, School of Medicine, University of Electronic Science and Technology of China, Chengdu, China; ^3^Department of Anesthesiology, West China Hospital, Sichuan University, Chengdu, China

**Keywords:** celiac plexus, neurolysis, cancer pain, pain management, pancreatic cancer, palliative care

## Abstract

**Introduction:**

Abdominal and back pain is the most frequent symptom in patients with pancreatic cancer, with pain management being extremely challenging. This study aimed to evaluate pain control, opioid consumption, pain-interfered quality of life, and survival after early and delayed computed tomography (CT)-guided celiac plexus neurolysis (CPN).

**Methods:**

A retrospective analysis of pancreatic cancer patients receiving CPN for pain (*n* = 56) between June 2018 and June 2021 was done. The patients were grouped as early group (*n* = 22) and delayed group (*n* = 34) on the basis of the presence of persistent refractory pain according to expert consensus on refractory cancer pain.

**Results:**

Both groups were comparable in demographic characteristics and baseline pain conditions measured using the numeric rating scale (5.77 ± 1.23 vs. 6.27 ± 1.21; *p* = 0.141). The pain scores were significantly reduced in both groups; early CPN resulted in significantly lower scores from 3 to 5 months. The opioid consumption gradually decreased to a minimum at 2 weeks but increased at 1 month (35.56 ± 30.14 mg and 50.48 ± 47.90 mg, respectively); significantly larger consumption from 2 to 4 months was seen in the delayed group. The total pain interference was lower than baseline in all patients, with significant improvement after early CPN in sleep, appetite, enjoyment of life, and mood. The average survival time of the two groups was comparable.

**Conclusion:**

Early application of CT-guided CPN for patients with advanced pancreatic cancer may help reduce pain exacerbation and opioids consumption, without influencing the survival.

## Introduction

Patients with pancreatic cancer frequently present with abdominal and back pain, with a reported incidence of 75% at the time of diagnosis and approximately 90% in patients with advanced staged cancer (1). Accordingly, optimal symptom control in the form of pain management is critical to improving the quality of life in these patients (2). Nevertheless, since the pain generation is due to multiple factors, such as perineural malignancy invasion, neurogenic inflammation, or ductal obstruction, pain control in patients with pancreatic cancer can be challenging (3, 4). Although the World Health Organization’s (WHO) analgesic ladder provides effective pain alleviation in over 70% of patients with general cancer, there are certain limitations to this strategy in cases of advanced pancreatic cancer because of frequent pain exacerbations (5, 6). Consequently, interventional procedures are considered the fourth step in the WHO analgesic policy (7).

Celiac plexus neurolysis (CPN) is a well-established verified technique for relieving pain, reducing opioid consumption, and improving cancer symptom burden for patients with abdominal malignancy (8–10). Nevertheless, this procedure is usually undertaken as the last resort for the management of refractory pain, which can decrease its effectiveness due to the high rate of neural invasion in pancreatic cancer (11, 12). The existing literature suggests that the severity of pain correlates strongly with perineural invasion and an adverse tumor microenvironment, which are both associated with poor prognosis (13–15). The recently emerging concepts of cancer pain management offer more promising analgesic strategies, among which, the modified WHO analgesic ladder prescribes a two-way path for the treatment of cancer pain—to start high and move backward down (16). Some clinicians advocate the application of CPN as a first-line treatment option to achieve better pain control for pancreatic cancer in a palliative situation (6). This is supported by multiple pieces of research which indicate that early palliative care for patients with cancer pain can result in improved quality of life and emotional status (17–19).

Nevertheless, whether early CPN is more beneficial than a delayed procedure in patients with advanced pancreatic cancer remains unclear. Thus, we conducted this retrospective cohort study to evaluate pain control, opioid consumption, pain-related quality of life, and survival in patients with advanced pancreatic cancer undergoing early and delayed CPN.

## Materials and methods

### Study design

We conducted an observational retrospective cohort analysis of the medical records obtained from a single oncology specialized hospital of patients with advanced pancreatic cancer, who suffered from moderate to severe pain and underwent CPN at the pain management department, from June 2018 to June 2021. All patients signed an informed consent before receiving the interventional procedure. The data collection and publication protocols were regulated by the Institutional Review Board.

Pain specialists in our department are responsible for providing comprehensive pain assessment, analgesic, and interventional treatment, as well as health follow-ups for cancer patients. Accordingly, patients experiencing pancreatic cancer pain were initially evaluated for their pain and previous analgesic strategies and then assigned to receive CPN after adequate preoperative optimization. Using a percutaneous antecrural approach under computed tomography (CT) guidance, a total of 6 mL of iohexol (Omnipaque) and lidocaine compounds was injected bilaterally into the target antecrural space. After confirming the spread of the contrast and successful test block, 20 mL of 100% ethanol was injected. The patients were then admitted for 24 h observation; after discharge, they continued to receive analgesic modulation and follow-up in our outpatient clinic.

During the follow-up, the nurse specialist evaluated the following items at fixed time intervals (baseline, 1 day, 1 week, 2 weeks,1 month, 2 months, 3 months, 4 months, 5 months and 6 months after operation)—pain score using the numerical rating scale (NRS), frequency of breakthrough pain recorded when happening ≥3 times/day, analgesic consumption converted into morphine equivalent, and pain inferred quality of life measured by the brief pain inventory (BPI). The BPI is used to assess eight items of functioning interfered by pain: general activity, mood, walking ability, normal work, relationship with others, sleep, appetite, and enjoyment of life. Each item is rated from 0 (does not interfere) to 10 (complete interference). The pain scores and analgesics consumption were re-evaluated up to 6 months postoperatively, as the neurolytic effects reportedly remain stable for approximately 3–6 months (20, 21). However, we were not able to assess pain interference with BPI in most patients 4 months after the procedure because of rapid disease progression.

### Participants and inclusion criteria

Data satisfying the following criteria was collected: (a) patients aged 18–80 years with advanced pancreatic cancer and received CPN at the pain management department; (b) having upper abdomen and/or middle back pain due to pancreatic cancer; (c) having NRS ≥4 points at baseline; (d) receiving analgesic medication and equivalent oral morphine ≥40 mg per day before procedure, and (e) having no invasion detected in the insertion path. Patients who were given further antitumor therapies or other interventions for pain control were excluded from the study.

### Early and delayed intervention: definition

Patients were divided into the early CPN group—to receive the operation as soon as they fulfilled the criteria at the index visit, or the delayed group—those who underwent a wait-and-see analgesic titration and received CPN only when they developed persistent refractory pain or with intolerable adverse effects. This definition is based on the expert consensus from the Committee of Rehabilitation and Palliative Care of China, which identifies refractory cancer pain based on the following two criteria: (a) persistent pain score ≥4 and/or breakthrough pain ≥3 times/day and (b) unsatisfactory pain relief after at least 2 weeks of standardized medication and/or causes intolerable side effects (22).

### Data collection and outcome measures

Demographic characteristics of all patients including age, gender, tumor classification, staging, course duration, comorbidities, and history of antitumor treatment were extracted from the hospital’s database. Data regarding pain score, adverse reactions, opioid consumption, and BPI pain inference were reviewed through previous follow-up records. Survival time data were obtained through the community health system before data analysis in December 2021.The sample size of the database study is primarily comprehensive, encompassing all the available data. However, a prior sample size calculation was conducted for scientific interpretation, focusing on the NRS scores at 3 months post-operation taking into account a potential dropout rate of 20%. This calculation aimed to provide 80% statistical power for detecting significant treatment differences, with a two-sided type 1 error set at 5% in a two-sample *t*-test. The outcome indicated the necessity of a sample size of 20 patients for each group.

As the primary parameter of interest, we performed a multidimensional assessment of the pain conditions, with respect to location, intensity, and breakthrough times. Subsequently, analgesic changes were analyzed with morphine equivalent conversion. To compare the overall pain interference between early and delayed CPN, a total BPI score (0–80 points) and that for the eight items individually were calculated.

### Statistical analysis

We used SPSS version 26.0 (IBM Inc., Armonk, NY, United States) to perform all analyses. NRS, BPI scores, and opioid consumption were presented as means with standard deviation. A two-sample *t-*test was used to compare the mean differences between continuous variables in the case of normally distributed data; otherwise, the Mann–Whitney *U* test was used for analysis. Breakthrough pain, tumor classification, staging, and complications were presented as frequency and percentages (%), and chi-square (*χ*^2^) or Wilcoxon rank-sum tests, as appropriate, were used for comparison between groups. Additionally, Kaplan–Meier survival curves were constructed for patient survival in months and compared using log-rank tests. A *p*-value of ≤0.05 was considered for statistical significance.

## Results

### Patient characteristics

**Figure 1:**
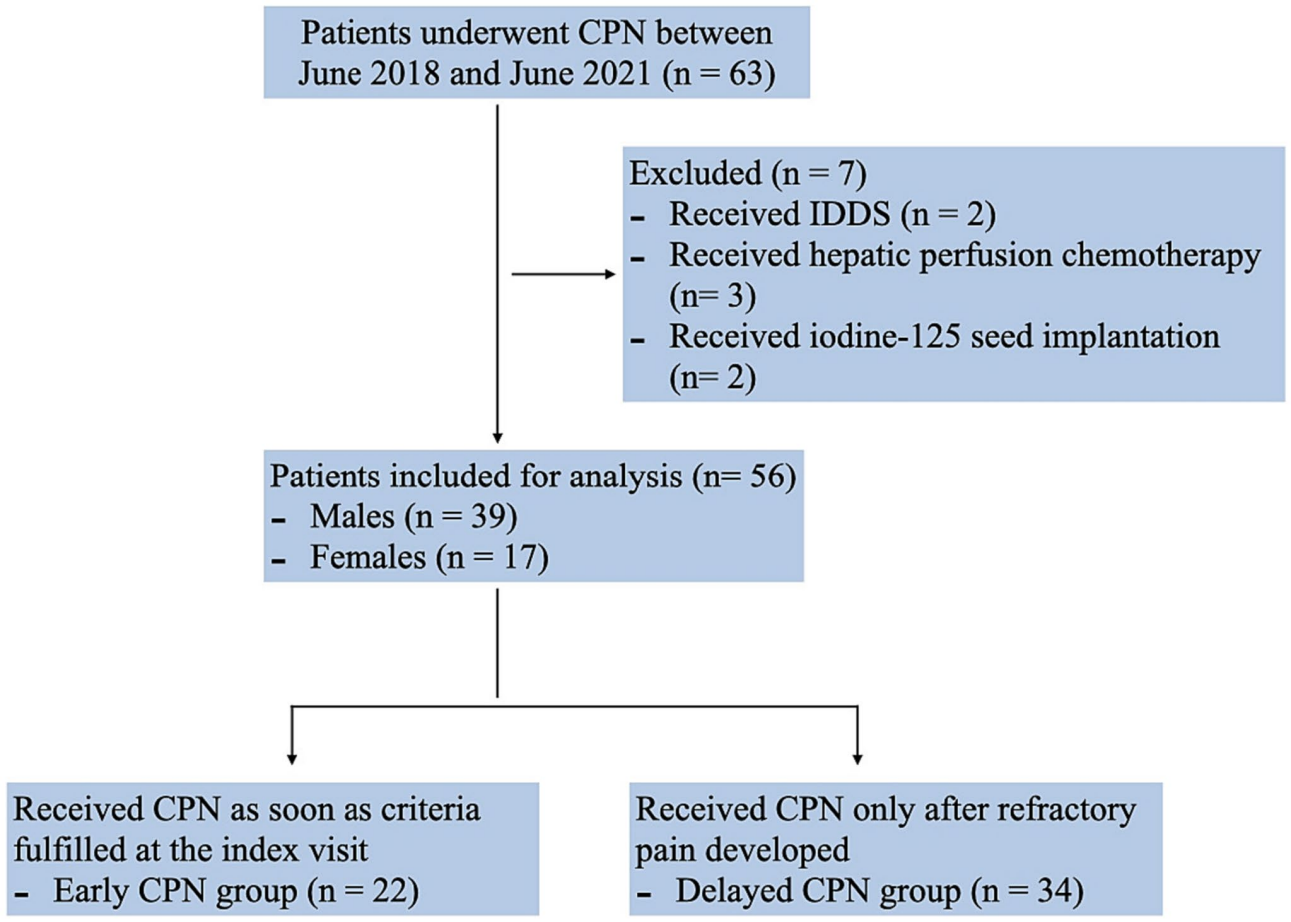
Flowchart of the subject selection.

A total of 63 patients with pancreatic cancer underwent CPN between June 2018 and June 2021 at our hospital. Of these, two patients were excluded since they were administered implantable drug delivery systems (IDDS) as a combined pain control solution. Three patients who received hepatic perfusion chemotherapy for metastases after CPN, and two who received iodine-125 seed implantation were also not included in the analysis. The final analysis included data for 56 patients (39 males and 17 females) (Figure 1). The majority of patients had cancer of the pancreatic body and tail (*n* = 21, 37.5%), followed by head adenocarcinoma (*n* = 19, 33.9%); 39 patients were at stage IV (69.6%), and 39 were in a malnutrition condition (69.6%). All patients had received multicourse oncology treatment before CPN. The time gaps between the first visit to receiving CPN were 3.45 (1.65) vs. 35.06 (9.98) days in the early and delayed group. Table 1 shows the basic clinical and demographic characteristics of the study population.

**Table 1:** Basic conditions of the study population.

	Early group	Delayed group	*p*-value
	*n* = 22 (%)	*n* = 34 (%)	
**Age (years)**			
Mean (SD)	56.64 (13.70)	56.53 (9.91)	0.973
**Gender**			
Male	15 (68.2)	24 (70.6)	0.848
Female	7 (31.8)	10 (29.4)	
**Pancreatic cancer location**			
Head	8 (36.4)	11 (32.4)	0.605
Body/tail	8 (36.4)	13 (38.2)	
Whole pancreas	1 (4.5)	5 (14.7)	
Metastatic pancreatic cancer	5 (22.7)	5 (14.7)	
**Time gap (days)**			
Mean (SD)	3.45 (1.65)	35.06 (9.98)	<0.001^a^
**Course duration (months)**			
Mean (SD)	5.59 (4.16)	4.79 (4.28)	0.314
**Cancer stages**			
IIB	2 (9.1)	1 (2.9)	0.598
III	5 (22.7)	9 (26.5)	
IV	15 (68.2)	24 (70.6)	
**Comorbidities**			
Hypertension	5 (22.7)	8 (23.5)	0.933
Diabetes	3 (13.6)	5 (14.7)	
COPD	0 (0.0)	1 (2.9)	
Osteoporosis	0 (0.0)	1 (2.9)	
Liver dysfunction	4 (18.2)	6 (17.6)	
Renal dysfunction	0 (0.0)	1 (2.9)	
Malnutrition	15 (68.2)	24(70.6)	
**Prior anti-tumor treatment**			
Surgery	5 (22.7)	4 (11.8)	0.839
Radiotherapy	6 (27.3)	6 (17.6)	
Intraoperative radiotherapy	7 (31.8)	11 (32.4)	
Chemotherapy	12 (54.5)	11 (32.4)	
Other treatments	11 (50.0)	16 (47.1)	

a Significance was detected between early and delayed group.

### Pain condition

**Figure 2:**
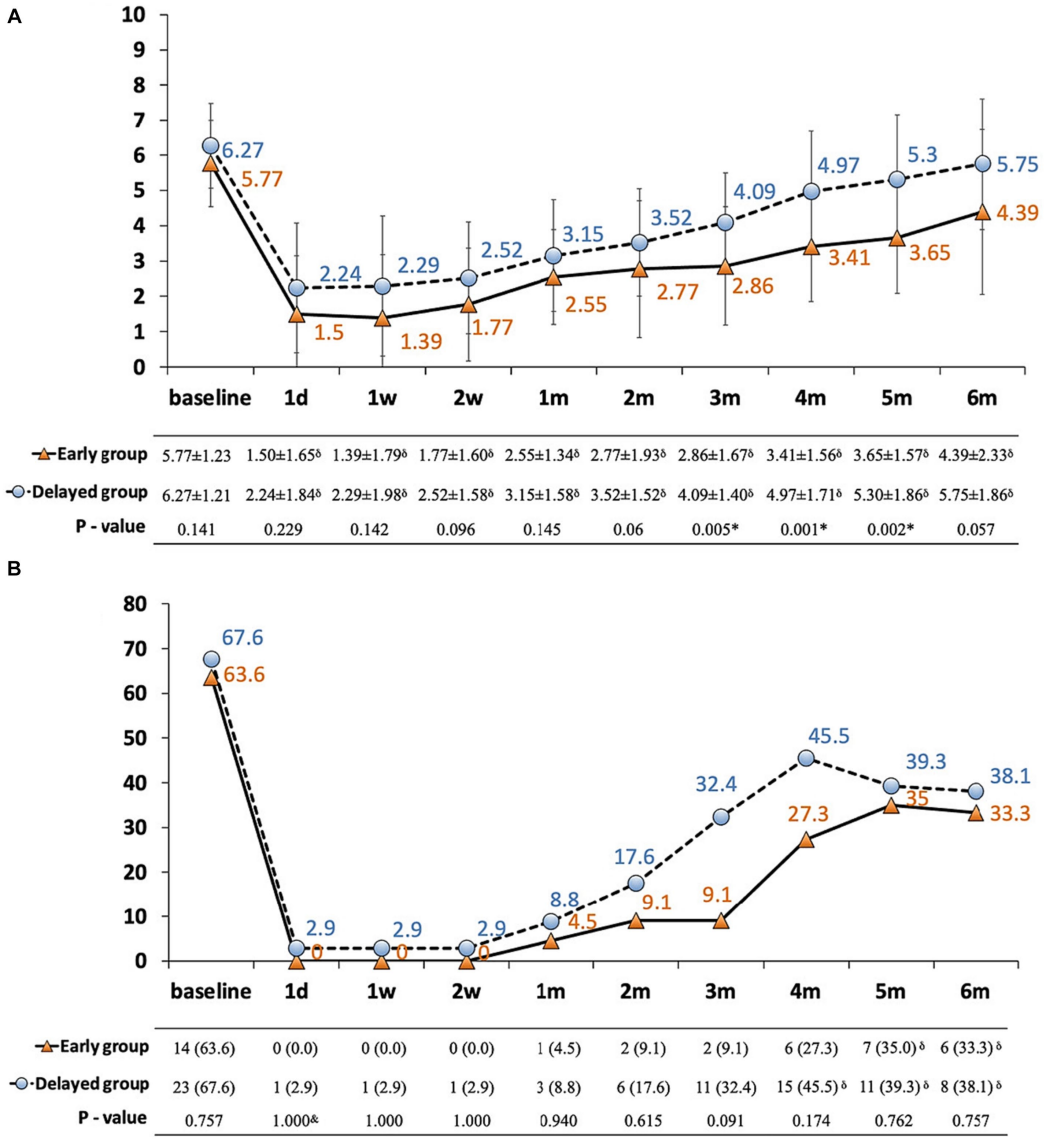
**(A,B)** Pain conditions in patients with pancreatic cancer between early and delayed CPN group. **(A)** NRS plotted against time; **(B)** incidence of breakthrough pain ≥3 times/day plotted against time. Solid line with orange triangles: early group; dashed line with blue dots: delayed group; thin bars represent standard error of the mean (SEM); ^*^significance was detected between early and delayed group; ^δ^significance was detected between follow-ups and baseline; NRS: numerical rating scale.

Patients in both groups reported experiencing moderate to severe pain, with a baseline NRS of 5.77 ± 1.23 and 6.27 ± 1.21, respectively. A significant reduction in NRS scores was observed in both groups after the procedure; however, a trend for a rebound was detected from 1 to 6 months. Nonetheless, those receiving early CPN presented with a lower pain score than the delayed group, and a significant difference was observed from 3 to 5 months (Figure 2A). Additionally, changes in the breakthrough pain corresponded with the NRS, demonstrating a significant decrease after CPN. Although the frequency of breakthrough pain was higher in the delayed group, no between-group difference was observed (Figure 2B).

### Analgesic consumption and adverse effects

The baseline equivalent oral morphine consumption was comparable in both groups, i.e., 78.89 ± 32.70 mg and 81.43 ± 48.09 mg, respectively. The analgesic consumption gradually reduced to a minimum at 2 weeks, followed by an increase in demand at 1 month (35.56 ± 30.14 and 50.48 ± 47.90 mg, respectively). Furthermore, patients in the delayed CPN group showed significantly greater opioid consumption from 2 to 4 months, and the increase in opioids started earlier compared with the early CPN group (Table 2). Both groups were statistically similar regarding CPN-related adverse reactions, the most frequent being dizziness (*n* = 12, 54.5% vs. *n* = 15, 44.1%), hypotension (*n* = 7, 31.8% vs. *n* = 11, 32.4%), and diarrhea (*n* = 7, 31.8% vs. *n* = 10, 29.4%) (Table 3). Additionally, one patient in the delayed group developed hematochezia on the second day after the operation, which was transient and resolved spontaneously after rehydration and fasting.

**Table 2:** Changes in opioids consumption after CPN.

	Early group	Delayed group	*p*-value
	Mean ± SD	Mean ± SD	
Baseline	78.89 ± 32.70	81.43 ± 48.09	0.905
1 day	48.89 ± 38.18^b^	57.62 ± 46.57^b^	0.617
1 week	36.67 ± 29.90^b^	53.33 ± 46.94^b^	0.320
2 weeks	35.56 ± 30.91^b^	50.48 ± 47.90^b^	0.267
1 month	35.56 ± 30.14^b^	56.19 ± 58.78^b^	0.188
2 months	41.82 ± 34.73^b^	74.12 ± 50.52^b^	0.011^a^
3 months	47.27 ± 30.73^b^	77.65 ± 55.38^b^	0.035^a^
4 months	53.33 ± 30.68^b^	81.90 ± 55.82	0.045^a^
5 months	62.22 ± 35.57^b^	90.48 ± 58.61	0.160
6 months	66.67 ± 35.65	96.19 ± 59.62^b^	0.162

a Significance was detected between early and delayed group.

b Significance was detected between follow-ups and baseline.

**Table 3:** Comparison of CPN related adverse reactions.

	Early group	Delayed group	*p*-value
	*n* = 22 (%)	*n* = 34 (%)	
Hypotension	7 (31.8)	11 (32.4)	0.967
Diarrhea	7 (31.8)	10 (29.4)	0.848
Dizziness	12 (54.5)	15 (44.1)	0.446
Headache	0 (0.0)	1 (2.9)	1.000
Localized pain	3 (13.6)	5 (14.7)	1.000
Nausea	5 (22.7)	6 (17.6)	0.902
Vomiting	0 (0.0)	1 (2.9)	1.000
Hematochezia	0 (0.0)	1 (2.9)	1.000

No differences were noted between groups.

### Pain-related quality of life and survival time

**Figure 3:**
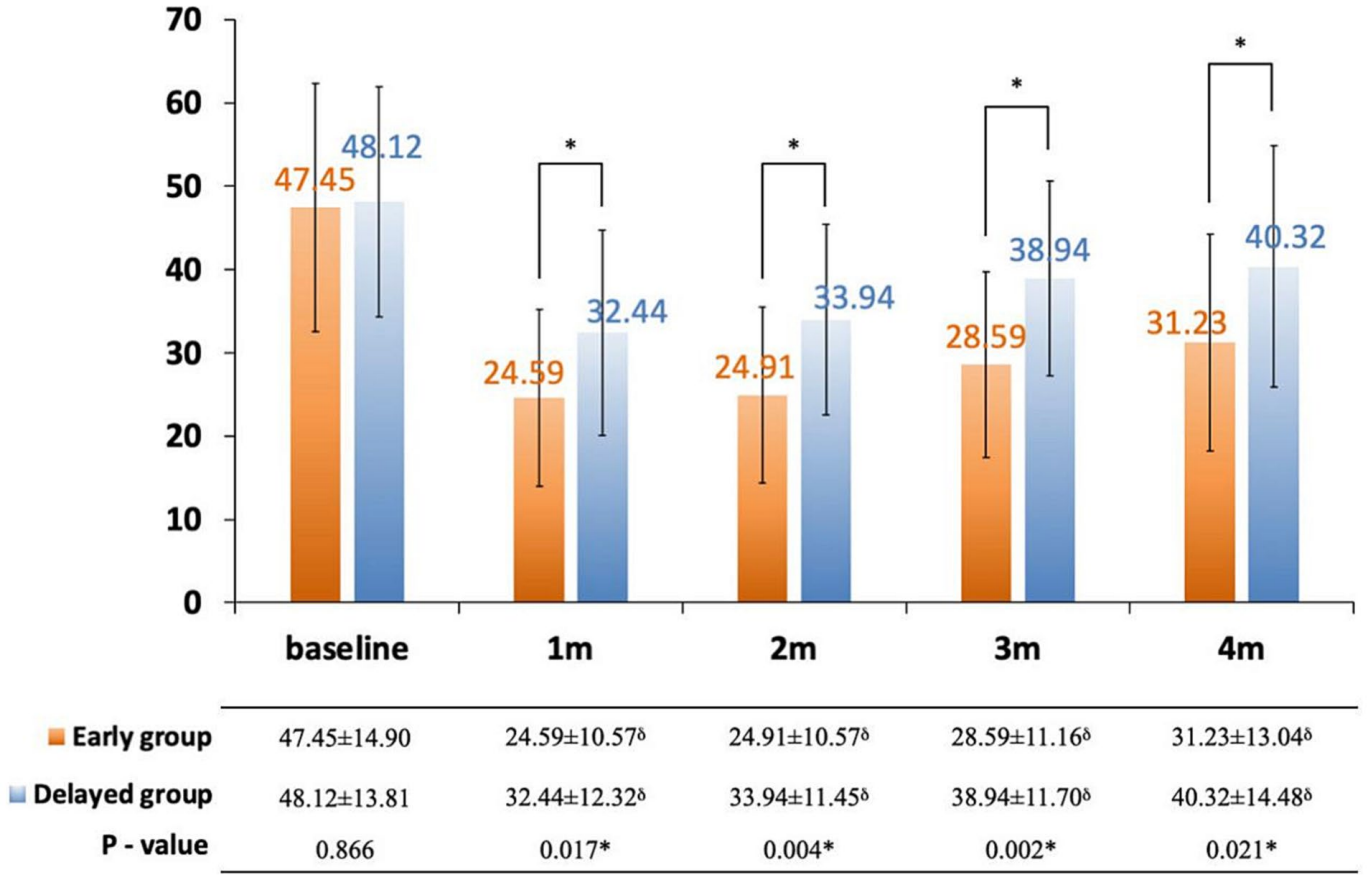
Change in total pain inference score between early and delayed group. Orange bars: early group; blue bars: delayed group; thin bars represent standard error of the mean (SEM); ^*^significance was detected between early and delayed group; ^δ^significance was detected between follow-ups and baseline.

**Figure 4:**
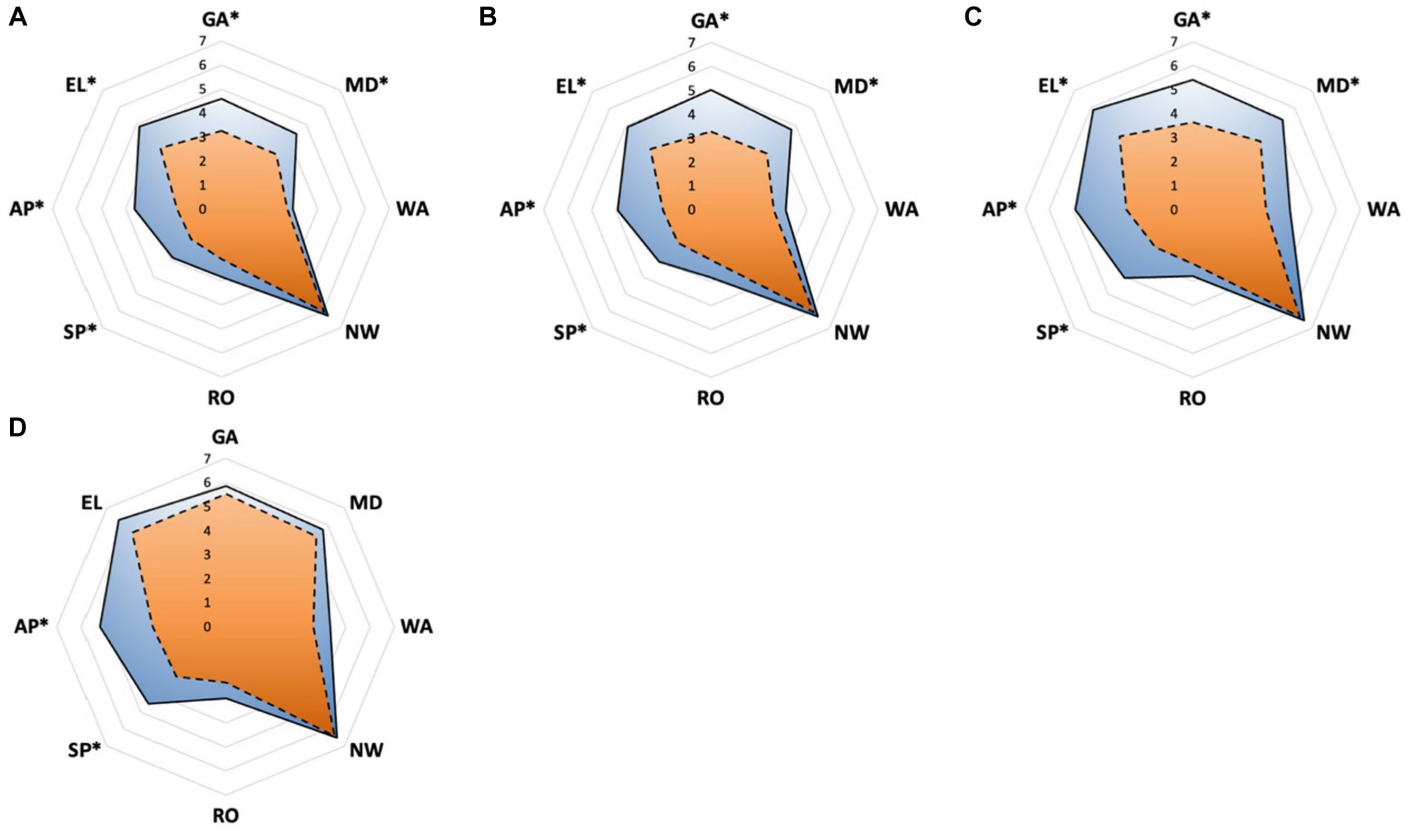
**(A–D)** Pain inference of eight sections measured by BPI between early and delayed group. **(A)** The domain scores at 1 month; **(B)** the domain scores at 2 months; **(C)** the domain scores at 3 months; **(D)** the domain scores at 4 months. Orange zone with dashed edge: early group; blue zone with solid edge: delayed group; GA, general activity; MD, mood; WA, walking ability; NW, normal work; RO, relationship with others; SP, sleep; AP, appetite; EL, enjoyment of life; ^*^significance was detected between early and delayed group.

**Figure 5:**
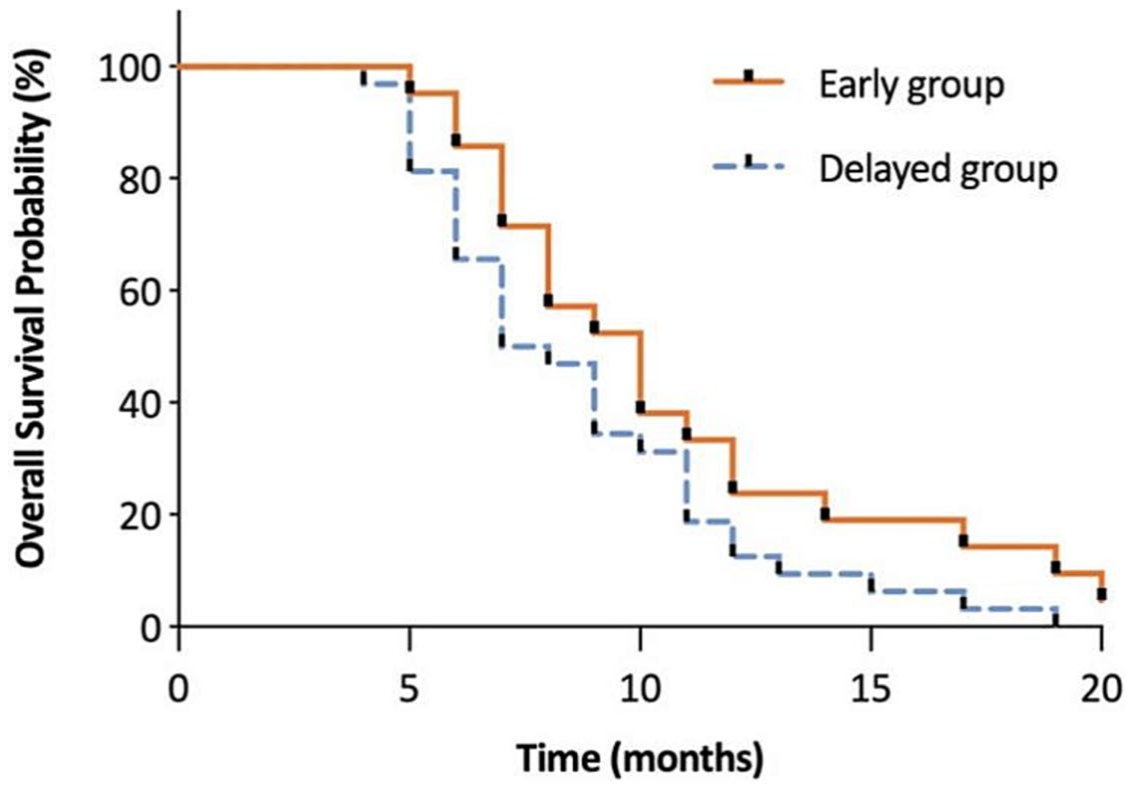
The evaluation of overall survival in months between early and delayed group. This figure shows the survival rates for both groups calculated by Kaplan–Meier survival curves. The average survival time was 11.18 and 8.75 months in the early and delayed CPN groups, respectively. No significant between-group difference was found using the log-rank test (*χ*^2^ = 2.501, *p* = 0.114).

The interference of pain with daily activities and emotional status was evaluated using BPI. The posttreatment total interference was significantly lower than baseline in all patients, suggesting that the overall health status was improved after CPN. Patients in the early group reported significantly low interference from 1 to 4 months posttreatment (Figure 3). Specifically, mood-related interference in the early group was significantly lower than in the delayed group, mainly evident in the “enjoyment of life” and “mood” items (Figures 4A–C). Meanwhile, early CPN also led to better sleep and appetite improvement (Figures 4A–D). However, no significant differences were detected in the activity-related interference (the average scores of work and walking), except for general activity (Figures 4A–C). Figure 5 shows the survival rates for both groups calculated by Kaplan–Meier survival curves. The average survival time was 11.18 and 8.75 months in the early and delayed CPN groups, respectively. No significant between-group difference was found using the log-rank test (*χ*^2^ = 2.501, *p* = 0.114).

## Discussion

The results of our study demonstrate that early CPN is beneficial in preventing pain progression and opioid consumption, besides improving the overall pain-interfered QoL. Pancreatic cancer is one of the most painful malignancies often associated with delayed diagnosis and the poorest prognosis rendering palliative pain management challenging for the patients (23). Chemical neurolysis is usually achieved by alcohol or phenol under imaging guidance, which can provide 3–6 months of pain relief for patients who have a limited life expectancy; moreover, substantial evidence suggests that, in contrast to general analgesia, CPN can provide even greater pain relief and result in reduced morphine consumption (21, 24, 25). However, many factors influence the effectiveness of pain relief from CPN, with retroperitoneal tumoral invasion being the most significant (26, 27). Therefore, patients delayed to CPN may experience relatively lower pain improvement. However, in most clinical cases, the patients with pancreatic cancer are under increasing opioids with unsatisfied pain relief. In this scenario, decisions between continued morphine titration or CPN have to be made. In our study we found that the time gap from the initial visit to the implementation of CPN could exceed 1 month between the two groups (3.45 days vs. 35.06 days), even though the diagnosis of refractory cancer pain could be made within 2 weeks. The parallel courses of opioid titration and disease progression may contribute to the delayed CNP decision. Recent studies support the application of a neurolytic procedure for pain control early after inadequate opioid therapy (28). Notably, a prospective study described using thoracoscopic splanchnicectomy as the first step of the analgesic ladder for pancreatic cancer pain and reported greater pain improvement, QoL, and longer survival time (12).

Although the pain scores were comparable between the two groups from 1 day to 2 months postoperatively, the early group was associated with significantly reduced NRS and the number of breakthrough pain from 3 to 5 months, suggesting a better prognosis with the early procedure. CPN aims to block nociceptive transmission raised from the celiac plexus, which is located anterior to the abdominal aorta and the celiac trunk (29). Accordingly, an adequate spread of the neurolytic solution into the preaortic space is one of the key determinants of the successful block (30). Conversely, perineural invasion into the extra-pancreatic nerve plexus is the most common pathologic characteristic of pancreatic cancer (31, 32). Depending on these factors, an optimal time window should be considered when administering CPN for patients with pancreatic cancer.

We observed that both early and delayed groups demonstrated decreased opioids consumption compared with baseline levels. To avert any withdrawal symptoms, the analgesic use was not reduced abruptly after the operation, with the lowest opioids consumption achieved by 2–4 weeks. Subsequently, the ongoing adjustment of opioids was increased in both groups, but the delayed group required an earlier rise and higher requirement. This outcome was not unexpected and was consistent with the pain progression, as well as the analgesic strategy we applied, i.e., sufficient opioids were administrated as required. Since CPN is not an isolated form of palliative pain management but a part of the broader analgesic strategy, opioids remain the mainstay for cancer pain control (33). Nonetheless, several studies have demonstrated that successful and timely neurolysis reduces the need for opioid consumption until the end of life (34–36). Furthermore, recent data suggest that CPN administration via endoscopic ultrasound at the time of diagnosis may help avert the increased opioid consumption spiral (24); our results further corroborate these results.

Expectedly, the overall pain interference in QoL exhibited a greater improvement in patients who received early CPN, notably in terms of sleep, appetite, and mood-related interference items. However, early CPN did not produce a prominent improvement in work and walking. It is known that patients with advanced cancer experience improved mood and QoL, require less aggressive end-of-life care, and have longer survival by integrating early supportive care (19, 37, 38). Furthermore, neurolysis for patients with abdominal malignancy is reported to improve both the QoL and longevity, which can probably be attributed to a reduction in opioid-related side effects (39). However, contrary to the above findings, a retrospective case–control study suggested that patients who underwent celiac neurolysis had shorter survival compared with the controls who did not (40). We observed that the survival times in both groups were comparable. In particular, the initial CA19-9 levels, tumor stage, and treatment intensity are all prognostic factors that influence the survival time of patients with advanced pancreatic cancer (41, 42). A recent study identified pancreatectomy with chemotherapy as a favorable prognostic factor for metastatic pancreatic cancer and developed a nomogram to predict 6 months, 12 months, and 18 months overall survival probabilities, considering factors such as age, tumor size, marital status, gender, and tumor grade (43). However, it remains indeterminate whether CPN itself is an independent factor for survival.

There were certain limitations of this study. Since it was a retrospective study, our findings are based on limited data collected from baseline to 6 months from a single institution. Additionally, the BPI scores were only available for the first 4 months and patients in the delayed group had a poorer response to prior oncologic therapy and pain control, both of which may have affected the outcome. In terms of grouping, the definition of early and delayed intervention was based on whether CNP was performed from the first visit or after a wait-and-see policy after at least 2 weeks of analgesic titration. However, we believe that a refined grouping method or subgroup analysis which considering tumor staging or time from diagnosis may help determine the overall pain course, but was not available in our database. Nevertheless, our study is the first to show the potential benefits of early application of CPN for pain control in patients with advanced pancreatic cancer, whom have experienced a poor response on moderate dose of opioids.

## Conclusion

The results of this retrospective cohort study suggest that early application of CT-guided CPN for patients with advanced pancreatic cancer offers multiple potential advantages, including reduced exacerbation of pain, reduced opioids consumption, and improved pain-interfered QoL; nonetheless, it is not associated with improved survival. To investigate the optimal procedure timing in different subgroups, a further large-scale randomized-controlled trial is required.

## Data availability statement

The original contributions presented in the study are included in the article/supplementary material, further inquiries can be directed to the corresponding author.

## Ethics statement

The studies involving humans were approved by Ethics Committee of the Sichuan Cancer Hospital (Approval number: SCCHEC-02-2022-069). The studies were conducted in accordance with the local legislation and institutional requirements. Written informed consent for participation was not required from the participants or the participants’ legal guardians/next of kin in accordance with the national legislation and institutional requirements.

## Author contributions

FL: Conceptualization, Data curation, Formal analysis, Investigation, Methodology, Project administration, Resources, Software, Supervision, Validation, Visualization, Writing – original draft, Writing – review & editing. XW: Data curation, Formal analysis, Investigation, Methodology, Project administration, Software, Validation, Visualization, Writing – review & editing. JT: Data curation, Formal analysis, Investigation, Methodology, Project administration, Resources, Software, Validation, Visualization, Writing – review & editing. XL: Conceptualization, Formal analysis, Funding acquisition, Methodology, Project administration, Resources, Supervision, Validation, Visualization, Writing – review & editing.
